# Minimum acceptable diet use and its associated factors among children aged 6–23 in Ghana: a mixed effect analysis using Ghana Demographic and Health Survey

**DOI:** 10.3389/fpubh.2024.1402909

**Published:** 2024-09-04

**Authors:** Berhan Tekeba, Belayneh Shetie Workneh, Alebachew Ferede Zegeye, Almaz Tefera Gonete, Gebreeyesus Abera Zeleke, Tadesse Tarik Tamir

**Affiliations:** ^1^Department of Pediatrics and Child Health Nursing, School of Nursing, College of Medicine and Health Sciences, University of Gondar, Gondar, Ethiopia; ^2^Department of Emergency and Critical Care Nursing, School of Nursing, College of Medicine and Health Sciences, University of Gondar, Gondar, Ethiopia; ^3^Department of Medical Nursing, School of Nursing, College of Medicine and Health Sciences, University of Gondar, Gondar, Ethiopia; ^4^Department of Surgical Nursing, School of Nursing, College of Medicine and Health Sciences, University of Gondar, Gondar, Ethiopia

**Keywords:** minimum acceptable diet, infant and young children feeding practice, 6–23 months determinants, Ghana Demographic and Health Survey, 2022

## Abstract

**Introduction:**

Inappropriate feeding practices are a major contributor to child malnutrition. To monitor the feeding practices of young children, current and frequent studies are required. However, as far as our searches are concerned, there is a scarcity of up-to-date information on attainment of the minimum acceptable diet and its predictors in the study area. Therefore, this study aimed to assess the magnitude of attainment of the minimum acceptable diet and its associated factors among children aged 6–23 in Ghana by using the most recent data.

**Methods:**

Secondary data analysis was conducted based on the demographic and health survey data conducted in Ghana in 2022. A total weighted sample of 2,621 children aged 6–23 months in the 5 years preceding the survey was included in this study. A multi-level logistic regression model was used to identify the determinants of the minimum acceptable diet. The adjusted odds ratio at 95% Cl was computed to assess the strength and significance of the association between explanatory and outcome variables. Factors with a *p*-value of <0.05 are declared statistically significant.

**Results:**

The national prevalence of the attainment of the minimum acceptable diet in Ghana was 26.40% (95% CI: 24.82–28.06). Child from mother with higher education (AOR = 1.96; 95% CI: 1.56–3.31) and father with higher education (AOR = 1.59; 95% CI: 1.04–2.41), Children having postnatal visit (AOR = 1.29; 95% CI: 1.03–1.62), being in the child age of 9–11 months (AOR = 2.09; 95% CI: 1.42–5.03) and 12–23 months (AOR = 3.62; 95% CI: 2.61–5.03), being in a middle (AOR = 1.66; 95% CI: 1.14–3.06), and rich wealth quintile (AOR = 2.06; 95% CI: 1.37–3.10), breastfed children (AOR = 3.30; 95% CI: 2.38–4.56), being in a high-community poverty (AOR = 0.65; 95% CI: 0.44–0.96), and being in the Savannah region (AOR = 0.32; 95% CI: 0.16–0.67) were factors significantly associated with the minimum acceptable diet use.

**Conclusion:**

Many children are still far behind in meeting the minimum acceptable diet in Ghana as per 90% of WHO-recommended coverage. Measures should be taken to optimize the minimum acceptable diet attainment in the country. Thus, policymakers, the government, and other relevant authorities should focus on the early initiation of complementary feeding, the Savannah region, further empowering women, and enhancing breast-feeding and household wealth status.

## Introduction

The minimum acceptable diet (MAD) is an indicator of infant and young infant child-feeding practice (1, 2) launched by the World Health Organization (WHO) and the United Nations International Children’s Emergency Fund (UNICEF), which is a combination of both the minimum meal frequency and the minimum dietary diversity. Minimum dietary diversity is a proxy for adequate micronutrient density in food and is defined as children who received five or more of the eight food groups (3, 4); whereas minimum meal frequency intake is a proxy for meeting energy requirements and is defined as daily consumption of ≥2 times for children aged 6–8 months, ≥3 times for those aged 9–23 months, and ≥ 4 times for those who were not breastfed (5, 6). A minimum acceptable diet as an indicator of infant and young child-feeding practice is a simpler, valid, and reliable method of assessing complementary feeding practice (7).

Malnutrition continues to be a major public health problem in low-and middle-income countries (8). Infants and young children are more susceptible to malnutrition, which commonly leads to child morbidity and death (9). Despite the WHO/UNICEF recommendations, most studies revealed a suboptimal rate of infant and young child-feeding practice globally (10). Analysis of the UNICEF global database report for 2024 revealed that only 21% of young children use MAD globally.^1^ A synthesis of national survey data from 80 low-and middle-income countries also revealed that only 10.1% of infants and young children received MAD (9). The attainment of MAD remains a significant challenge for developing countries, particularly in sub-Saharan Africa. Ghana, as a sub-Saharan African country, is experiencing a burden of undernutrition.^2^ One important contributing factor to malnutrition in Ghana is inappropriate feeding practices after 6 months of age (11). Identifying potential risk factors is a crucial step toward improving infant and young children’s feeding practices in Ghana.

During the first 2 years of life or the child’s 1,000 days of life, inadequate feeding practices have a negative effect on children’s development and health. It has been determined that this time frame is the “critical window” for promoting a child’s optimal growth, health, and development. Inappropriate infant and young child-feeding (IYCF) during this period results in a significant threat to child health by impairing cognitive development, compromised educational achievement, and low economic productivity, which become difficult to reverse later in life (5, 12, 13). Although it is not usually a direct cause (7), inappropriate feeding practices during the first 2 years of life were responsible for more than two-thirds of malnutrition-related deaths (14–17). Stunted growth, limited cognitive development, and micronutrient deficiency result from inappropriate feeding practices (18, 19). To overcome undernutrition, for children who are 6 months and older, complementary feeding needs to be started (5, 12). An estimated 6% of mortality among children under five could be avoided by providing appropriate complementary feeding. Thus, concerned bodies should strive to attain minimum acceptable diet use in low-income sub-Saharan African countries, including Ghana.

As reviewed in prior literature, both individual and community-level factors are responsible for attainment of the minimum acceptable diet among young children aged 6–23 months. Accordingly, child age, child sex, birth order, plurality of birth, maternal and paternal education and occupation, wealth index, ANC visit, PNC visit, media exposure, and institutional delivery were individual-level determinants of MAD. Whereas, place of residence and regional variations account for community-level determinants (20–27).

Some countries in Africa, including Ghana, have made marginal improvements in reducing malnutrition among under-five children. However, according to the GDHS 2022 report, 18, 7, and 12% of under-five children were stunted, wasted, and underweight, respectively (21) and 59% of children do not meet the minimum diversity requirement.^3^ Thus, there is a need to overcome undernutrition and improve the attainment of dietary requirements to improve young child-feeding practices. The government of Ghana, in collaboration with stakeholders, stands to reduce undernutrition, including the Ghana Nutrition Improvement Program Project, the National Nutritional Policy, School Feeding Programs, and the Start Right Feed right from birth to 2 years of life (28). Despite this, a large proportion of children are unable to attain infant and young child-feeding practices in Ghana (10).

Suboptimal infant and young children feeding practices were a major contributor to undernutrition in young children (29). Nutritional improvement through appropriate complementary feeding practices is critical for young children’s healthy growth and development (30, 31). Thus, the nutritional status of children, particularly youngsters, requires current and frequent studies to monitor their feeding practices. In addition, identifying potential predictors that lower the attainment of MAD use helps concerned bodies prioritize appropriate interventions. As far as the investigator’s search of the literature is concerned, there is a paucity of recently updated information on child attainment of the minimum acceptable diet among young children aged 6–23 in Ghana. Therefore, this study aimed at assessing the magnitude of attainment of the minimum acceptable diet and its predictors among children aged 6–23 in Ghana by using the most recent data, which will help to inform relevant authorities and identify potential barriers against attainment of the minimum acceptable diet.

## Method and materials

### Study setting

The study setting is the Republic of Ghana. The Republic of Ghana is one of the countries in West Africa and has a total area of 238,533 km^2^ (32). Its borders are to the north with Burkina Faso, to the east with Togo, to the south by the Atlantic Ocean, and to the west with Côte d’Ivoire. The nation currently consists of 16 regions. The following 16 regions constitute Ghana: Greater Accra area, Central, Eastern, Upper East, Upper West, Volta, Northern, and Ashanti. The other regions include Brong, Oti, Ahafo, Bono East, North East, Savannah, Western North, and Western.

### Data source and sampling procedure

This study was done using the data extracted from the standard Ghana Demographic and Health Survey 2022. The data were collected from October 2022 to January 2023, and it is the seventh DHS series. This study used the most recent DHS dataset. Every 5 years, nationwide surveys known as the DHS are conducted worldwide in low-and middle-income nations. Data were collected from a nationally representative sample of approximately 18,450 households from all 16 regions in Ghana. The sampling procedure used in the 2022 GDHS was stratified two-stage cluster sampling, designed to yield representative results at the national level in urban and rural areas and for most DHS indicators in each country’s region.

In the first stage, 618 targeted clusters were selected from the sampling frame using a probability-proportional-to-size strategy for urban and rural areas in each region. Then the number of targeted clusters was selected with equal probability through systematic random sampling of the clusters selected in the first phase for urban and rural areas.

In the second stage, after the selection of clusters, a household listing and map updating were carried out in all of the selected clusters to develop a list of households for each cluster. The list served as a sampling frame for the household sample. A fixed number of 30 households in each cluster were randomly selected from the list for interviews. Thus, 17,993 households were successfully interviewed in 618 clusters. Of the 15 households interviewed, 10,014 women ages 15–49 and 7,044 men ages 15–59 completed individual interviews. For this study, women with a birth history who had given birth 5 years before the surveys were included. This study included a sample of 2,621 kids between the ages of 6 and 23 months for analysis. Only the last births of women aged 15–49 years preceding the survey were included in the study (see Footnote 3).

### Inclusion criteria

All children aged 6–23 months preceding the survey years in the selected EAs who were in the study area were included in the study.

### Exclusion criteria

Children in the age category of 6–23 months, which is not assessed for MAD based on DHS guidelines and has a missing value for the outcome variables, were excluded.

### Study population

**Figure 1:**
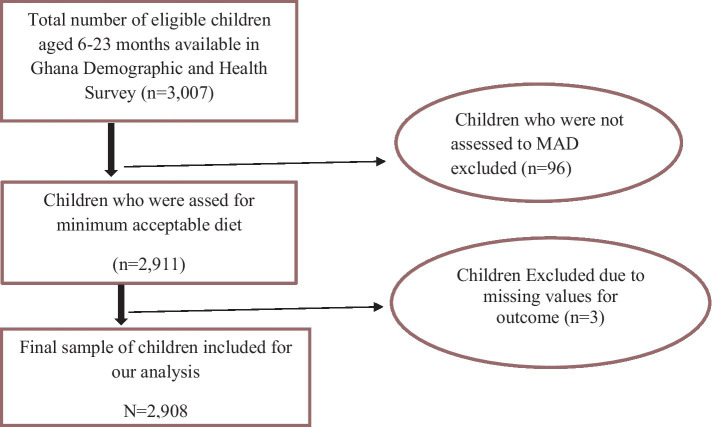
Diagrammatic representation of sample size determination of the minimum acceptable diet among children aged 6–23 months in Ghana, GDHS 2022. MAD, minimum acceptable diet.

The study population consisted of children aged 6–23 months preceding the 5-year survey period in the selected enumeration areas, which are the primary sampling units of the survey cluster. The mother or caregiver was interviewed for the survey in the country, and mothers who had more than one child during the survey period were asked about the most recent child (Figure 1).

### Study variables

#### Outcome of variable

##### Minimum acceptable diet

The outcome variable is the minimum acceptable diet, which is the sum of minimum meal frequency and dietary diversity for breast-feeding children and minimum breast-feeding frequency for non-breast-feeding children. If the child attained the above indicators, the child was considered yes (“1”) for attaining the minimum acceptable diet and no (“0”) for not attaining the minimum acceptable diet.

##### Dietary diversity

Dietary diversity was used to assess the proportion of children 6–23 months of age who have consumed at least five out of eight pre-defined foods the previous day or night. The eight food groups are: (i) breast milk; (ii) grains, white/pale starchy roots, tubers, and plantains; (iii) legumes and nuts; (iv) dairy products (infant formula, milk, yogurt, cheese); (v) flesh foods (meat, fish, poultry, and liver/organ meats); (vi) eggs; (vii) vitamin A-rich fruit and vegetables; (viii) other fruits and vegetables (33).

##### Minimum meal frequency

Minimum meal frequency (MMF) was used to assess the percentage of breastfed and non-breastfed children aged 6–23 months who eat solid and semi-solid foods, including milk for non-breastfed infants, the minimum number of times in a day (34). It was calculated for breastfed children aged 6–23 months as eating at least twice for children aged 6–8 months, three times for children aged 9–23, and four times for non-breastfed children.

##### Minimum milk feeding frequency

It was used to assess the frequency of milk feeding for non-breast-feeding children aged 6–23 months; if the child consumed at least two milk feedings during the previous day or night, it was considered as attaining minimum milk feeding frequency (33).

#### Independent variables

Both individual-and community-level factors were reviewed from different literatures, and these include child age and sex, maternal education, working status and educational status, parent (husband) education and employment status, ANC visit, media exposure, counseling on breast-feeding, place of delivery, number of household members and under-five children, household head sex, wealth index, birth order, PNC visit, and mothers involvement in household decision-making. Whereas, whether distance to a health facility is a problem or not, residence, region, community women’s illiteracy level, community poverty level, and community media exposure were community-level variables aggregated from individual-level factors.

The wealth index was re-categorized as poor, middle, and rich (35). Maternal age was re-categorized into 15–19, 20–34, and 35–49 (36). ANC use was classified as optimal, and non-optimal (37).

Postnatal care was categorized as “yes” and “no.” Birth order was categorized into (first order, second to fourth order, and fifth and above order). Child age was categorized into 6–8 months, 9–11 months, and 12–23 months. Maternal education status was categorized as no education, primary, secondary, and higher. Media exposure was categorized into “yes” and “no” (34). Distance to a health facility was categorized as “a big problem” or “not a big problem.” Mother’s involvement in healthcare decision-making was categorized as “yes” and “no.” Pregnancy wontedness was categorized as “yes” and “no.” Place of delivery was categorized as “home delivery” and “health facility delivery.” Current marital status was categorized as “currently married” and “currently unmarried.” Plurality refers to multiple pregnancies and is categorized as “single” and “multiple.”

Family size was categorized as below five (<5) and above or equal to five (≥5). Number of under-five children categorized into “one,” “two,” and “three and above.” Child sex and household head sex were classified as “male” and “female.” Current breastfeeding was categorized as “yes” for currently breast-feeding children and “no” for not currently breast-feeding children.

#### Community-level variables

Community-level women’s illiteracy and community-level poverty were aggregated based on individual women’s characteristics of education and wealth index, respectively. Since the aggregate values for all generated variables have no meaning at the individual level, they were categorized into groups based on median values. Median values were used to categorize as high and low because all aggregated variables were not normally distributed. Similar procedures were applied to all aggregate variables.

### Model building for multi-level analysis

DHS data exhibit nested dependencies, where individuals are nested within communities. It employs stratified, two-stage cluster sampling. Clusters (communities) are sampled, and within each cluster, households and individuals are further selected. We conducted a likelihood ratio test, comparing ordinary logistic regression to multilevel logistic regression. The results affirmed a significant improvement when using multilevel models, reinforcing their suitability. In summary, multilevel analysis of DHS data unveils the intricate web of nested dependencies, allowing us to disentangle individual and contextual effects.

Thus, using the traditional logistic regression model violates the assumptions of independent observation and equal variance across clusters. Therefore, a multi-level logistic regression analysis was employed in this study in order to account for the hierarchical nature of DHS data. A bivariate multi-level logistic regression model was employed in the study to identify the variables associated with the attainment of the minimum acceptable diet. In the analysis, four models were fitted. The analysis was based on weighted sample to mitigate the effect of any sample imbalance. The first (null) model contains only the outcome variables to test random variability and estimate the intra-cluster correlation coefficient (ICC). The second model contains individual-level variables; the third model contains only community-level variables; and the fourth model contains both individual-level and community-level variables (38). Due to the hierarchical nature of the model, models were compared using deviation = −2 (log-likelihood ratio), and the best-fit model was determined by taking the model with the lowest deviance. The variance inflation factor (VIF) was used to detect multicollinearity, and the mean value of VIF of final model was 1.46. Regarding covariate selection and model building, initially, important variables considered in prior literature were selected, and a chi-square test was done between each covariate and outcome. Then, covariates that had a *p*-value of <0.05 were selected for bivariate analysis. Secondly, a covariate that had a p-value of less than 0.2 was considered for multilevel multivariable analysis. Finally, covariates that had *p*-values <0.05 in multivariable analysis were declared statistically significant. The details of the bivariate analysis are found in Supplementary Table S1.

### Parameter estimation method

#### The fixed effects (a measure of association)

They were used to estimate the association between the likelihood of the prevalence of attainment of MAD and explanatory variables at both individual and community levels. The association between dependent and independent variables was assessed, and its strength has been presented using an adjusted odds ratio (AOR) and 95% confidence intervals with a *p*-value <0.05. Hence, the log of the probability of feeding MAD was modeled using a two-level multilevel by using the Stata syntax xtmelogit (39):


$$\mathrm{logit}\;\left(\pi ij\right)=\log\;\left[\pi ij/\left(1-\pi ij\right)\right]=\beta 0+\beta 1xij+\beta 2xij\ldots +u0j+e0ij\text{,}$$

where *πij*: the probability of the *i*th young children attaining MAD (1 − *πij*), the probability of young children not attaining MAD *β*0: intercept, *βn*: regression coefficient indicating that a unit increase in *x* can cause *β*n unit increase in the probability of attainment of MAD, *Xij*: independent variables *u*0*j*: community-level error (the effect of community on the mother’s decision to provide MAD), *e*0*ij*: individual-level errors. The clustered data nature and the within-and between-community variation were taken into account, assuming each community has a different intercept (*βn*) and fixed coefficient (*β*0) (40).

#### Random effects (a measure of variation)

Variation of the outcome variable or random effects was assessed using the proportional change in variance (PCV), intra-class correlation coefficient (ICC), and median odds ratio (MOR) (41, 42).

The ICC shows the variation in attainment of the minimum acceptable diet due to community characteristics, which was calculated as: ICC = σ2/(σ2 + π^2^/3), where σ^2^ is the variance of the cluster (43). The higher the ICC, the more relevant the community characteristics are for understanding individual variation in the attainment of the minimum acceptable diet.

MOR is the median value of the odds ratio between the areas with the highest attainment of a minimum acceptable diet and the area with the lowest attainment of a minimum acceptable diet when randomly picking out two younger children from two clusters, which was calculated as: MOR = e^0.95√ σ2^, where σ^2^ is the variance of the cluster. In this study, MOR shows the extent to which the individual probability of attainment of the minimum acceptable diet is determined by the residential area (44).

Furthermore, the PCV illustrates how different factors account for variations in the prevalence of attainment of the minimum acceptable diet and is computed as PCV = (V_null_ − V_c_)/V_null_, where V_c_ is the cluster-level variance and V_null_ is the variance of the null model (45).

### Ethics statement and consent to participate

The authors analyzed secondary, publicly available data obtained from the DHS program database. There was no additional ethical approval, and informed consent was obtained by the authors. In order to perform our study, we registered with the DHS web archive, requested the dataset, and were granted permission to access and download the data files. According to the DHS report, all participant data were anonymized during the collection of the survey data. More details regarding DHS data and ethical standards are available online at http://www.dhsprogram.com.

## Results

### Socio-demographic, health service utilization, behavioral and community-level factors of the minimum acceptable diet among children aged 6–23 months in Ghana

This study was conducted among a weighted sample of 2,621 young children (6–23 months) in Ghana. More than half (52.96%) of respondents live in rural areas. More than two-thirds (61.14%) of mothers were married. More than two-thirds (67.57%) of children were aged 12–23 months. More than two-thirds (69.73%) of mothers were aged 20–34 years old. More than half (64.45%) of mothers completed their secondary school education and above. More than three-fourths (78.56%) of mothers were working at the time of the survey period; on the other hand, the majority (94.47%) of husbands or parents were employed. The majority (89.19%) of women had an optimal ANC visit, or more than two-thirds (83.48%) of mothers had media exposure. More than half (59.82%) of households had a large (above 5) family size. Nearly three-fourths (70.89%) of households were headed by men. The majority (87.34%) of mothers were involved in their household decisions. The majority (90.16%) of children born were wanted. Three-fourths (75.95%) of mothers got counseling on breast-feeding (Table 1).

**Table 1:** Socio-demographic, behavioral, and health service utilization, and community-level characteristics of children aged 6–23 months in Ghana, GDHS 2022.

Variable		Total weighted frequency (%)	Minimum acceptable diet		*p*-value
			Yes [*N* (%)]	No [*N* (%)]	
Mothers’ age	15–19	153 (5.84)	32 (20.98)	121 (79.02)	0.005
	20–34	1827 (69.73)	510 (27.9)	1,317 (72.05)	
	35–49	640 (24.43)	186 (29.09)	454 (70.91)	
Marital status	Married	1,602 (61.14)	440 (27.42)	289 (28.37)	0.886
	Unmarried	1,019 (38.89)	1,162 (72.53)	730 (71.63)	
Mothers’ education	No education	556 (21.21)	111 (20.01)	444 (79.99)	0.000
	Primary	401 (15.30)	91 (22.75)	310 (77.25)	
	Secondary	1,422 (54.25)	416 (29.24)	1,006 (70.76)	
	Higher	242 (9.23)	111 (45.77)	131 (54.23)	
Mothers’ working status	Yes	2059 (78.56)	601 (29.19)	1,458 (70.81)	0.000
	No	561 (21.40)	128 (22.78)	433 (77.22)	
Fathers’ education	No education	474 (21.54)	90 (18.93)	384 (81.07)	0.000
	Primary	225 (10.23)	64 (28.53)	161 (71.47)	
	Secondary	1,168 (53.09)	311 (26.59)	857 (73.41)	
	Higher	341 (15.50)	139 (41.72)	194 (58.28)	
Father employed	Yes	2,476 (94.47)	687 (27.74)	1789 (72.26)	0.75
	No	144 (5.49)	42 (29.11)	102 (70.89)	
Family size	<5	1,052 (40.14)	316 (30.02)	736 (69.98)	0.145
	≥5	1,568 (59.82)	413 (26.34)	1,155 (73.66)	
Number of under-five children	1	1,203 (46.0)	383 (31.86)	819 (68.14)	0.003
	2	1,042 (39.76)	271 (26.04)	771 (73.96)	
	≥3	376 (14.34)	74 (19.8)	301 (80.2)	
Household head sex	Male	1858 (70.89)	469 (25.25)	1,389 (74.75)	0.004
	Female	763 (29.11)	260 (34.08)	503 (65.92)	
Wealth index	Poor	1,164 (44.41)	26 (22.47)	903 (77.53)	0.000
	Middle	547 (20.87)	133 (24.35)	413 (75.65)	
	Rich	909 (34.68)	334 (36.75)	575 (63.25)	
Child age	6–8	438 (16.71)	68 (15.49)	370 (84.51)	0.000
	9–11	411 (15.68)	125 (30.5)	286 (69.5)	
	12–23	1771 (67.57)	536 (30.24)	1,236 (69.76)	
Child sex	Male	1,335 (50.93)	350 (26.24)	985 (73.76)	0.078
	Female	1,285 (49.06)	379 (29.46)	906 (70.54)	
Birth order	First	745 (28.42)	224 (30.08)	521 (69.92)	0.733
	Second–fourth	1,300 (49.60)	365 (28.1)	934 (71.90)	
	Fifth and above	576 (21.98)	140 (24.26)	436 (75.74)	
Currently breast-feeding	Yes	558 (21.3)	608 (29.46)	121 (21.77)	0.000
	No	2063 (78.7)	1,455 (70.54)	436 (78.23)	
Plurality	Single	2,493 (95.12)	707 (28.36)	1786 (71.64)	0.003
	Multiple	128 (4.88)	22 (17.29)	106 (82.71)	
Media exposure	Yes	2,188 (83.48)	638 (29.17)	1,550 (70.83)	0.000
	No	432 (16.48)	91 (21.0%)	341 (79.0%)	
ANC visit	Non-optimal	272 (10.87)	51 (18.7)	221 (81.3)	0.006
	Optimal	2,232 (89.17)	676 (30.3)	1,555 (69.7)	
PNC visit	Yes	1,306 (52.18)	418 (31.98)	888 (68.02)	0.003
	No	1,197 (47.82)	309 (25.84)	888 (74.16)	
Counseling on breast-feeding	Yes	1901 (75.95)	575 (30.27)	1,326 (69.73)	0.004
	No	603 (24.09)	152 (25.18)	451 (74.82)	
Mother involved in decision	Yes	1,273 (88.34)	370 (29.04)	903 (70.96)	0.683
	No	168 (11.66)	40 (24.12)	127 (75.88)	
Pregnancy wanted	Yes	2,363 (90.16)	651 (27.55)	1712 (72.45)	0.847
	No	258 (9.84)	78 (30.26)	180 (69.74)	
Place of delivery	Home	336 (12.82)	73 (21.73)	263 (78.27)	0.015
	Health facility	2,285 (87.18)	656 (28.71)	1,629 (71.29)	
Place of residence	Urban	1,233 (47.04)	395 (32.07)	837 (67.93)	0.000
	Rural	1,388 (52.96)	334 (24.04)	1,054 (75.96)	
Distance to health facility	Big problem	660 (25.18)	162 (24.59)	497 (75.41)	0.006
	No problem	1961 (74.82)	567 (28.9)	1,394 (71.1)	
Community poverty level	High	1,612 (61.50)	482 (29.92)	1,129 (70.08)	0.001
	Low	1,009 (38.50)	247 (24.46)	762 (75.54)	
Community women’s illiteracy	High	1759 (67.11)	534 (30.38)	195 (22.59)	0.008
	Low	862 (32.89)	1,224 (69.62)	667 (77.41)	
Community media exposure	High	1862 (71.04)	548 (29.45)	1,313 (70.55)	0.004
	Low	759 (28.96)	181 (23.81)	578 (76.19)	
Region	Western	159 (6.07)	50 (31.31)	110 (68.69)	0.000
	Central	281 (10.72)	103 (36.76)	178 (63.24)	
	Greater Accra	294 (11.22)	104 (35.3)	190 (64.7)	
	Volta	108 (4.12)	31 (28.33)	77 (71.67)	
	Eastern	176 (6.72)	37 (21.08)	139 (78.92)	
	Ashanti	490 (18.70)	133 (27.1)	357 (72.9)	
	Western north	73 (2.80)	19 (26.5)	54 (73.5)	
	Ahafo	56 (2.14)	14 (25.48)	42 (74.52)	
	Bono	86 (3.28)	21 (25.13)	64 (74.87)	
	Bono east	145 (5.53)	29 (19.76)	116 (80.24)	
	Oti	82 (3.13)	24 (29.25)	58 (70.75)	
	Northern	307 (11.71)	59 (19.23)	248 (80.77)	
	Savannah	75 (2.86)	9 (11.49)	66 (88.51)	
	North east	77 (2.94)	29 (38.07)	48 (61.93)	
	Upper east	131 (5.0)	44 (33.55)	87 (66.45)	
	Upper west	81 (3.09)	23 (28.34)	58 (71.66)	

ANC, antenatal care; PNC, postnatal care.

### Prevalence and factors associated with attainment of the minimum acceptable diet in Ghana (GDHS, 2023)

**Figure 2:**
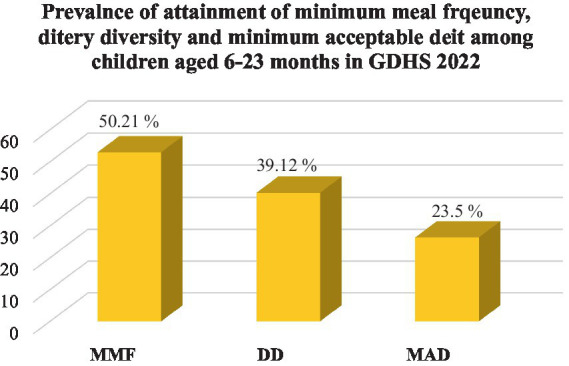
The national prevalence of minimum meal frequency, dietary diversity, and minimum acceptable diet of children aged 6–23 months in Ghana, GDHS 2022. MMF, minimum meal frequency; DD, dietary diversity; MAD, minimum acceptable diet.

According to this study, the attainment of the minimum acceptable diet among children aged 6–23 months in Ghana was 23.50% (95% CI: 22.12–26.07). The prevalence of dietary diversity and meal frequency was 39.12% (95% CI: 37.12–41.23) and 50.21 (95% CI: 48.95.14–52.17), respectively (Figure 2).

### Regional prevalence

**Figure 3:**
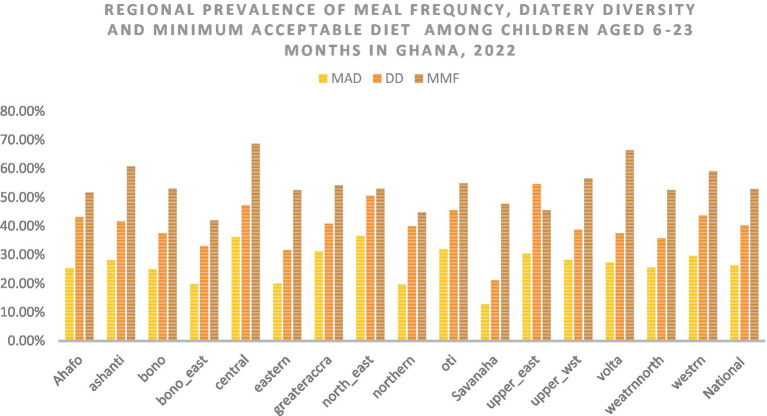
The overall, and regional prevalence of the minimum acceptable diet, dietary diversity, and meal frequency in Ghana. MAD, minimum acceptable diet; DD, dietary diversity; MMF, minimum meal frequency.

The highest prevalence of the minimum acceptable diet (34.5%), dietary diversity (53.2%), and minimum meal (67.14%) frequencies were observed in the North-East, Upper-East, and Central regions of Ghana, respectively. The lowest minimum acceptable diet (11.13%) and dietary diversity (20.02%) were observed in the Savanah region, and the lowest minimum meal frequency (40.06%) was observed in the Bono East region of Ghana (Figure 3).

### Random effect analysis and model comparison

The null model was run to determine whether the data supported assessing randomness at the community level. The ICC value in the null model indicates that 14.9% of the attainment of the minimum acceptable diet was due to the difference between clusters. In the null model, the odds of attainment of the minimum acceptable diet were 1.97 times variable between high and low clusters (heterogeneous among clusters). Regarding the final model PCV, about 43.1% of the variability in attainment of the minimum acceptable diet was attributed to both individual and community-level factors. Model III was selected as the best-fitting model since it had the lowest deviance (Table 2).

**Table 2:** Individual- and community-level variance for multilevel random intercept logit model predicting feeding practice of children aged 6–23 according to the minimum acceptable diet use in Ghana, GDHS 2022.

Parameter	Null model	Model I	Model II	Model III
Variance	0.58	0.57	0.34	0.33
ICC	14.99%	14.77%	9.4%	9.12%
MOR	1.97	2.05	1.74	1.74
PCV	Reference	17.24%	41.38%	43.1%
LLR	−1,620.32	−1,276.11	−1,591.63	−1,251.45
Deviance	3,240.64	2,552.22	3,183.26	2,502.9

ICC, intra-cluster correlation coefficient; MOR, median odds ratio; PCV, proportional change in variance; LLR, log-likelihood ratio.

### Factors associated with the minimum acceptable diet among children aged 6–23 in Ghana

In the final model (model III) of multivariable multilevel logistic regression, child age, household head sex, maternal and paternal education level, breast-feeding children, middle and richer wealth index, high-community poverty level, and Savanah region were factors significantly associated with the minimum acceptable diet use.

Accordingly, young children aged 9–11 and 12–23 were two (AOR = 2.09; 95% CI: 1.4–3.06) and three times (AOR = 3.62; 95% CI: 2.61–5.03) more likely to attain MAD as compared to young children aged 6–8 months. Similarly, children in middle and richer households were 1.66 (AOR = 1.66; 95% CI: 1.54–2.43) and 2 times (AOR = 2.06; 95% CI: 1.37–3.10) more likely to attain MAD, respectively, as compared to young children in poor households. Children born to a mother and father with higher education were 1.96 (AOR = 1.96; 95% CI: 1.56–3.31) and 1.59 times (AOR = 1.59; 95% CI: 1.04–2.41) more likely to use MAD as compared to children with parents with no formal education. Children who had a postnatal visit had 29% higher odds (AOR = 1.29; 95% CI: 1.03–1.62) of receiving the minimum acceptable diet as compared to their counterparts. Currently, breast-feeding young children are three times more likely (AOR = 3.30; 95% CI: 2.38–4.56) to attain MAD as compared to non-breast-feeding children. On the other hand, children from high-community poverty-level households had 35% reduced odds (AOR = 0.65; 95% CI: 0.44–0.96) of receiving MAD as compared to children from low-community poverty-level households. Similarly, young children who reside in the Savannah region were 68% less likely (AOR = 0.32; 95% CI: 0.16–0.67) to use the MAD as compared to children who reside in the western region of Ghana (Table 3).

**Table 3:** Multi-variable multilevel logistic regression analysis result of both individual-level and community-level factors associated with minimum acceptable diet use among young children aged 6–23 months in Ghana, GDHS 2022.

Variable	Response	Model I AOR (95% CI)	Model II AOR (95% CI)	Model III AOR (95% CI)
Socio-demographic-related factors				
Mothers’ age	15–19	0.56 (0.28–1.11)		0.54 (0.27–1.06)
	20–34	1		1
	35–49	1.16 (0.91–1.47)		1.17 (0.93–1.49)
Mothers’ education	No education	1		1
	Primary	1.10 (0.77–1.57)		1.11 (0.77–1.60)
	Secondary	1.20 (0.87–1.66)		1.24 (0.89–1.74)
	Higher	1.93 (1.15–3.24)		1.96 (1.16–3.31)*
Mothers’ working status	Yes	1		1
	No	0.82 (0.62–1.08)		0.84 (0.64–1.11)
Fathers’ education	No education	1		1
	Primary	1.31 (0.88–1.93)		1.32 (0.90–1.95)
	Secondary	1.04 (0.76–1.43)		1.03 (0.75–1.41)
	Higher	1.58 (1.04–2.42)		1.59 (1.04–2.41)*
Number of under five	1	0.85 (0.61–1.19)		0.87 (0.62–1.21)
	2	0.96 (0.70–1.33)		0.98 (0.71–1.34)
	≥5	1		1
Household head sex	Male	0.81 (0.62–1.05)		0.63 (0.44–1.97)
	Female	1		1
Wealth index	Poor	1		1
	Middle	1.33 (0.97–1.83)		1.66 (1.14–2.43)*
	Rich	1.67 (1.21–2.32)		2.06 (1.37–3.10)*
Child-related factors				
Child age	6–8	1		1
	9–11	2.07 (1.40–3.05)		2.09 (1.42–3.06)*
	12–23	3.56 (2.56–4.95)		3.62 (2.61–5.03)*
Counseling on breast-feeding	Yes	1.12 (0.86–1.46)		1.13 (0.87–1.48)
	No	1		1
Plurality	Single	0.79 (0.43–1.46)		0.80 (0.44–1.48)
	Multiple	1		1
Health utilization and behavioral-related factors				
Media exposure	Yes	1		1
	No	0.82 (0.61–1.11)		0.85 (0.63–1.15)
ANC visit	Optimal	1		1
	Non-optimal	0.84 (0.57–1.22)		0.83 (0.57–1.21)
PNC visit	Yes	1.28 (1.02–1.61)		1.29 (1.03–1.62)*
	No	1		1
Currently breast-feeding	Yes	3.23 (2.34–4.46)		3.30 (2.38, 4.56)*
	No	1		1
Place of delivery	Home	1		1
	Health facility	1.05 (0.74–1.50)		1.07 (0.75–1.53)
Distance difficulty	Big problem	0.97 (0.75–1.25)		0.97 (0.76–1.25)
	Not big problem	1		1
Community-level factors				
Place of residence	Urban		1.37 (1.06–1.77)	1.19 (0.88–1.61)
	Rural		1	1
Community poverty level	High		0.94 (0.70–1.26)	1.65 (0.44–0.96)*
	Low		1	1
Community women’s illiteracy	High		1.12 (0.85–1.47)	0.89 (0.65–1.23)
	Low		1	1
Region	Western		1	1
	Central		1.34 (0.75–2.39)	1.62 (0.83–3.15)
	Greater Accra		0.96 (0.51–1.79)	0.86 (0.42–1.75)
	Volta		0.89 (0.47–1.67)	0.86 (0.42–1.75)
	Eastern		0.56 (0.29–1.10)	0.53 (0.25–1.13)
	Ashanti		0.91 (0.51–1.62)	0.86 (0.44–1.66)
	Western north		0.82 (0.44–1.55)	0.70 (0.34–1.42)
	Ahafo		0.83 (0.44–1.56)	0.76 (0.37–1.56)
	Bono		0.80 (0.42–1.52)	0.64 (0.31–1.34)
	Bono east		0.59 (0.32–1.08)	0.56 (0.28–1.11)
	Oti		1.23 (0.67–2.23)	1.15 (0.59–2.26)
	Northern		0.66 (0.36–1.18)	0.52 (0.27–1.01)
	Savannah		0.37 (0.19–0.71)	0.32 (0.16–0.67)*
	North east		1.56 (0.86–2.80)	1.38 (0.72–2.67)
	Upper east		1.17 (0.65–2.11)	0.85 (0.44–1.64)
	Upper west		1.04 (0.58–1.88)	0.81 (0.42–1.56)

ANC, antenatal care; PNC, postnatal care.

*Statistically significant (*p*-value < 0.05).

Null model: This model contains only the outcome variables to test random variability and estimate the intra-cluster correlation coefficient (ICC).

Model I: This model contains individual-level variables that affect the outcome (MAD).

Model II: This model contains only community-level variables.

Model III: This model contains both individual and community-level variables.

Minimum acceptable diets were aggregated based on minimum meal frequency and dietary diversity.

Participants were categorized into two groups: those who attain a minimum acceptable diet (“yes”) and those who do not attain a minimum acceptable diet (“no”).

Covariates include individual-level factors like maternal and paternal education, age, sex, working status, antenatal and postnatal care, and so on. Community-level variables include community illiteracy level, community poverty level.

## Discussion

Inadequate infant and young child-feeding practices are the most important global problems and the major determinates of undernutrition. To attain optimal growth and development in the first 2 years of life, young children need adequate nutrition. Identifying and reducing the disabling factors behind adequate nutrition in this critical window period is a crucial step toward improving children’s overall health and wellbeing (31, 46). To the best of our knowledge, this study is based on the most recent data to determine the magnitude and independent predictors of MAD use among children aged 6–23 months in Ghana. Thus, it provides up-to-date evidence for relevant authorities to improve the feeding practices of infants and young children in Ghana.

This study revealed that 26.40% (95% CI: 24.82–28.06) of young children aged 6–23 attain the minimum acceptable diet in Ghana. This study’s findings were lower than the studies conducted in Guinea (28%) (47), Nepal (33%) (48), Indonesia (61.8%) (49), and Bangladesh (34.7%) (50). The discrepancy might be due to geographic variation, population growth and density, and socio-economic status. Additionally, food preparation, dietary restrictions, food item selection, meal scheduling, cultural beliefs, availability and accessibility of food, and childcare practices may differ across countries (51).

However, this study found higher than the UNICEF 2022 report, studies done in East Africa (11.56%), Ethiopia (11.3%) (52), West African countries (3–11) (53), sub-Saharan Africa (9.89%), Gambia (3.2%), Rwanda (21.4%) (54), Tanzania (15.9%) (55), previous studies in Ghana (11.72%) (7), Philippines (6.7%), and India (10.5%) (56–59).^4^ The possible explanation could be that our study, based on the most recent data, suggested that other countries might have made improvements like Ghana but have not yet reported. In addition, some of the sub-Saharan countries were at the lowest economic level as compared to Ghana’s lower-middle-income economy, since the economy had a direct impact on the nutritional status of the family, like increasing the purchasing ability of variety and quality food items for young children’s and whole households, where better nutrition might be available to the family (60). Moreover, Ghana nowadays has a mature and stable democracy, and strong media further helps citizens attain nutritional requirements by increasing productivity. Furthermore, there was a regional and global commitment to nutrition in Ghana that might bring further improvements to young children’s feeding practices in the country.^5^ Thus, other low-income countries shall take the strategy, initiatives, and nutritional interventions used in Ghana to enhance the feeding practices of young children.

Using multi-level logistic regression analysis, the key predictors of the minimum acceptable diet were child age, household head sex, maternal and paternal education level, breast-feeding children, middle and richer wealth index, high-community poverty level, and Savanah region.

Compared to children aged 6–8 months, children aged 9–11 months and 12–23 months had higher odds of minimum acceptable diet use. This indicates that as the child gets older, there is a high probability of attaining the minimum acceptable diet. This is supported by the studies done in different parts of the world (9, 11, 61). The possible explanation could be that mothers introduced other varieties of diets in addition to breast-feeding as the child grew older, or a late initiation of complementary feeding. In addition, mothers may perceive young children’s inability to digest foods like solid, semi-solid, and soft foods like meat, eggs, fruit, and vegetables due to the unsuitability of specific food items. Some societies in the world also consider not giving some food items and animal sources of food before 12 months of age and the eruption of teeth (62–64). So, it is important to advise mothers with younger children to start complementary feeding after the baby turns 6 months old during their follow-up and postnatal checks (65, 66).

Children in the middle and richer wealth quintiles had reduced odds of the minimum acceptable diet use as compared to children residing in the poor wealth quintile. This also indicates that as the family wealth status improved, the nutritional status of children improved. This supports the study done in the Philippines and Ethiopia (52, 59). The possible explanation could be that mothers from the richest households were more likely to give their children highly nutritious food as compared to mothers from poorer households, who were more likely to focus on quantity aspects of food (67). Poor households may lack the purchasing power to provide a sufficiently varied diet to feed their children. In addition, children from households with higher incomes have better resources to meet the MAD than their counterparts from the poorest households since they are exposed to better complementary feeding practices, resulting in improved nutrient adequacy and sufficiency (59). However, it is possible to get a diversified, feasible, and healthy diet at an affordable cost using locally available foods (68, 69).

Children born from parents with higher education had a higher chance of attaining the minimum acceptable use as compared to children born from uneducated parents. This study aligns with other studies done in different parts of the world (51, 57, 70, 71). This is due to the fact that as the mother’s education level grows, her knowledge, attitude, and practice may improve. Furthermore, higher education will provide nutritional counseling and enhance child-feeding practices (13, 72). Thus, educating parents is one key strategy to improve the nutritional status of young children.

Children who received postnatal care (PNC) were more likely to meet the minimum acceptable diet than their counterparts. This is supported by other studies (12, 73). One possible explanation could be that PNC checkups allow healthcare practitioners to personalize their counseling and assistance for mothers. By addressing specific issues, dispelling myths, and providing individualized guidance on feeding habits and dietary needs, clinicians help moms make informed decisions that encourage a minimum acceptable diet for their children (22).

Children from high-poverty communities are less likely to receive the minimal recommended diet than their contemporaries. The outcomes of this investigation were consistent with prior research (59, 74). In impoverished communities, various interconnected variables contribute to the challenge of providing appropriate and healthy food for children. These include restricted financial resources, limited resources for making nutritious meals, limited storage space, cultural influences, and food preferences (75, 76). Thus, the focus of relevant bodies should be on reducing income inequality and poverty.

Children who reside in the Savannah region of Ghana have a reduced use of the minimum acceptable as compared to children residing in the Western region of Ghana. This is supported by the previous study done in the country, as there is variation in the nutritional status of children that is difficult to explain, but cultural practices of the respective region might be the cause of the variations (11). Moreover, water scarcity, land degradation, and drought are major threats to the area, with desert-prone areas being particularly hard hit. In addition, it is the most unstable location due to erratic and unexpected rainfall, which reduces crop productivity and raises the risk of food shortages, malnutrition, and related issues (77, 78).

This study also found that currently breastfed children were more likely to attain meal frequency as compared to non-breast-fed children. This is supported by the studies done in different parts of the world (67, 79). The possible explanation could be that the new minimum acceptable diet indicator incorporates breast milk as one of the components of food items that help breast-feeding children attain minimum meal frequency, dietary diversity, and the minimum acceptable diet as a whole. For instance, the Ethiopian DHS 2016 report showed that the proportion of feeding according to the minimum acceptable dietary standard is somewhat lower among non-breast-feeding children than breast-feeding children (80). Moreover, as the study done in China revealed, extended breast-feeding is associated with improved nutritional status as measured by standard anthropometric indicators (79). However, a study done in India found that extended breast-feeding (6–23 months) does not show any significant difference in impact on the anthropometric measurements of child health.

This study’s strength was the use of multi-level modeling to make meaningful inferences and findings while accounting for the clustering effect in GDHS. The study has the potential to assist programmers and policymakers in developing effective national interventions because it is based on data from a countrywide survey. However, the study has its own limitations. Due to the cross-sectional nature of the study design, the results of this study could not infer a causal relationship between outcome and independent variables. In addition, recall bias may exist in this study due to the cross-sectional nature of DHS and its reliance on respondents’ self-reports, like 24-h recall of food intake by children, which might also be affected by social desirability bias, and the frequency and diversity of the food intake may be overestimated. Moreover, prior studies also revealed inconsistent findings of MAD attainment in Ghana from 2000 until now; thus, further temporal studies showing trends should be done. Finally, due to the DHS nature of the data, important social, political, agricultural, and cultural variables are not available. Thus, future research incorporating multiple causes of the attainment of the minimum acceptable feeding practice will be done.

Even though Ghana had improvement in minimum acceptable diet use as compared to previous reports. However, many children are still far behind in receiving the minimum acceptable diet in Ghana as per the WHO-recommended standard of 90% coverage (11). Therefore, measures should be taken further to optimize MAD utilization in the country. The study also found that factors at the individual and community levels were associated with the attainment of a minimum acceptable diet. The main factors that determined the minimum acceptable diet in our study were child age, household head sex, current breast-feeding, wealth index, and region. Therefore, interventions to improve MAD use should be implemented at the individual and community levels. Thus, government policymakers and relevant authorities should give special attention to the Savannah region, male-headed households, early initiation of complementary feeding, improving breast-feeding, and improving household wealth status in Ghana.

The government of Ghana should plan and work in short terms through the program that endorses awareness creation for male-headed households to give attention to children’s feeding practices, early initiation of complementary feeding, and improving breast-feeding. Long-term plans are also needed for the Savanah region and individual-level household wealth quintiles of the country to improve infant and young child-feeding practices.

## Data Availability

The datasets presented in this study can be found in online repositories. The names of the repository/repositories publicly available online at (http://www.dhsprogram.com). The datasets used and/or analyzed during the current study will available from the corresponding author on reasonable request.

## Footnotes

^1^ https://www.who.int/data/nutrition/nlis/info/infant-and-young-child-feeding

^2^ https://www.usaid.gov/sites/default/files/2022-05/Copy_of_Ghana-Nutrition-Profile_1.pdf

^3^ https://dhsprogram.com/pubs/pdf/PR149/PR149.pdf

^4^ https://data.unicef.org/topic/nutrition/diets/

^5^ https://2017-2020.usaid.gov/sites/default/files/documents/1864/Ghana-Nutrition-Profile-Mar2018-508.pdf

## References

[1] Statistics CBO. Nepal multiple indicator cluster survey 2019, survey findings report. Kathmandu, Nepal: Central Bureau of Statistics and UNICEF Nepal (2020).

[2] Sapkota S Thapa B Gyawali A Hu Y. Predictors of minimum acceptable diet among children aged 6–23 months in Nepal: a multilevel analysis of Nepal multiple Indicator cluster survey 2019. Nutrients. (2022) 14:3669. doi: 10.3390/nu14173669, PMID: 36079926 PMC9460334

[3] World Health Organization. Global nutrition monitoring framework: Operational guidance for tracking progress in meeting targets for 2025. Geneva: World Health Organization (2017).

[4] World Health Organization. Infant and young child feeding. Fact sheet no 342. Recuperado de (2014). Available at: http://www.who.int/mediacentre/factsheets/fs342/en

[5] Molla M Ejigu T Nega G. Complementary feeding practice and associated factors among mothers having children 6–23 months of age, Lasta District, Amhara region, Northeast Ethiopia. Adv Public Health. (2017) 2017:1–8. doi: 10.1155/2017/4567829

[6] World Health Organization. Indicators for assessing infant and young child feeding practices: Part 2: measurement. Geneva: World Health Organization (2010).

[7] Bain LE Awah PK Geraldine N Kindong NP Siga Y Bernard N. Malnutrition in Sub–Saharan Africa: burden, causes and prospects. Pan Afr Med J. (2013) 15, 120. doi: 10.11604/pamj.2013.15.120.2535 PMC3830470 24255726

[8] Gebremedhin S Baye K Bekele T Tharaney M Asrat Y Abebe Y. Predictors of dietary diversity in children ages 6 to 23 mo in largely food-insecure area of south Wollo, Ethiopia. Nutrition. (2017) 33:163–8. doi: 10.1016/j.nut.2016.06.002 27499206

[9] Acharya A Pradhan MR Das AK. Determinants of minimum acceptable diet feeding among children aged 6–23 months in Odisha, India. Public Health Nutr. (2021) 24:3834–44. doi: 10.1017/S1368980021002172 34034833 PMC10195256

[10] Akanbonga S Hasan T Chowdhury U Kaiser A Akter Bonny F Lim IE. Infant and young child feeding practices and associated socioeconomic and demographic factors among children aged 6–23 months in Ghana: findings from Ghana multiple Indicator cluster survey, 2017–2018. PLoS One. (2023) 18:e0286055. doi: 10.1371/journal.pone.0286055 37294773 PMC10256209

[11] Issaka AI Agho KE Burns P Page A Dibley MJ. Determinants of inadequate complementary feeding practices among children aged 6–23 months in Ghana. Public Health Nutr. (2015) 18:669–78. doi: 10.1017/S1368980014000834, PMID: 24844532 PMC10271301

[12] Gizaw G Tesfaye G. Minimum acceptable diet and factor associated with it among infant and young children age 6–23 months in North Shoa, Oromia region, Ethiopia. Int J Hom Sci. (2019) 5:1:1. doi: 10.11648/j.ijhnm.20190501.11

[13] Beyene M Worku AG Wassie MM. Dietary diversity, meal frequency and associated factors among infant and young children in Northwest Ethiopia: a cross-sectional study. BMC Public Health. (2015) 15:1–9. doi: 10.1186/s12889-015-2333-x 26433689 PMC4592571

[14] Kabir I Khanam M Agho KE Mihrshahi S Dibley MJ Roy SK. Determinants of inappropriate complementary feeding practices in infant and young children in Bangladesh: secondary data analysis of demographic health survey 2007. Matern Child Nutr. (2012) 8:11–27. doi: 10.1111/j.1740-8709.2011.00379.x, PMID: 22168516 PMC6860519

[15] World Health Organization. Guiding principles for complementary feeding of the breastfed child. Geneva: World Health Organization (2003).

[16] Black RE Allen LH Bhutta ZA Caulfield LE De Onis M Ezzati M. Maternal and child undernutrition: global and regional exposures and health consequences. Lancet. (2008) 371:243–60. doi: 10.1016/S0140-6736(07)61690-0 18207566

[17] Black RE Morris SS Bryce J. Where and why are 10 million children dying every year? Lancet. (2003) 361:2226–34. doi: 10.1016/S0140-6736(03)13779-8, PMID: 12842379

[18] Molla A Egata G Getacher L Kebede B Sayih A Arega M. Minimum acceptable diet and associated factors among infants and young children aged 6–23 months in Amhara region, Central Ethiopia: community-based cross-sectional study. BMJ Open. (2021) 11:e044284. doi: 10.1136/bmjopen-2020-044284 PMC8112428 33972337

[19] Frempong RB Annim SKJH. Dietary diversity and child malnutrition in Ghana. Heliyon. (2017) 3:e00298. doi: 10.1016/j.heliyon.2017.e00298 28503669 PMC5419825

[20] Herman H Mansur AR Chang Y-J. Factors associated with appropriate complementary feeding: a scoping review. J Pediatr Nurs. (2023) 71:e75–89. doi: 10.1016/j.pedn.2023.04.017, PMID: 37150632

[21] Jeyakumar A Babar P Menon P Nair R Jungari S Medhekar A. Determinants of complementary feeding practices among children aged 6–24 months in urban slums of Pune, Maharashtra, in India. J Health Popul Nutr. (2023) 42:1–13. doi: 10.1186/s41043-022-00342-6 36658658 PMC9850568

[22] Feleke FW Mulaw GF. Minimum acceptable diet and its predictors among children aged 6-23 months in Mareka District, southern Ethiopia: community based cross-sectional study. Int J. (2020) 9:203. doi: 10.1111/mcn.13647

[23] Ahoya B Kavle JA Straubinger S Gathi CM. Accelerating progress for complementary feeding in Kenya: key government actions and the way forward. Matern Child Nutr. (2019) 15:e12723. doi: 10.1111/mcn.12723, PMID: 30748122 PMC6594063

[24] Ogbo FA Page A Idoko J Claudio F Agho KE. Trends in complementary feeding indicators in Nigeria, 2003–2013. BMJ Open. (2015) 5:e008467. doi: 10.1136/bmjopen-2015-008467, PMID: 26443657 PMC4606380

[25] Mulat E Alem G Woyraw W Temesgen H. Uptake of minimum acceptable diet among children aged 6–23 months in orthodox religion followers during fasting season in rural area, DEMBECHA, north West Ethiopia. BMC Nut. (2019) 5:1–10. doi: 10.1186/s40795-019-0274-y PMC7050747 32153931

[26] Adekanmbi VT Kayode GA Uthman OA. Individual and contextual factors associated with childhood stunting in Nigeria: a multilevel analysis. Matern Child Nutr. (2013) 9:244–59. doi: 10.1111/j.1740-8709.2011.00361.x, PMID: 22004134 PMC6860873

[27] Kebede M. Minimum acceptable diet practice and its associated factors among Children’s aged 6–23 months in rural communities of Goncha District, North West Ethiopia, 2020. BMC Nutr. (2020) 7:40. doi: 10.1186/s40795-021-00444-0 PMC8290531 34281613

[28] Aurino E Gelli A Adamba C Osei-Akoto I Alderman H. Food for thought?: experimental evidence on the learning impacts of a large-scale school feeding. Program. (2023) 58:74–111. doi: 10.3368/jhr.58.3.1019-10515R1

[29] Kandala N-B Madungu TP Emina JB Nzita KP Cappuccio FP. Malnutrition among children under the age of five in the Democratic Republic of Congo (DRC): does geographic location matter? BMC Public Health. (2011) 11:1–15. doi: 10.1186/1471-2458-11-261 21518428 PMC3111378

[30] World Health Organization. Strengthening action to improve feeding of infants and young children 6-23 months of age in nutrition and child health programmes: Report of proceedings, Geneva, 6-9 October 2008. Geneva: World Health Organization (2008).

[31] Marriott BP White A Hadden L Davies JC Wallingford JC. World health organization (WHO) infant and young child feeding indicators: associations with growth measures in 14 low‐income countries. Matern Child Nutr. (2012) 8:354–70. doi: 10.1111/j.1740-8709.2011.00380.x 22171937 PMC6860880

[32] Ghana Statistical Service. 2010 population & housing census: National analytical report: Ghana statistics service. Accra: Ghana Statistical Service (2013).

[33] World Health Organization. Indicators for assessing infant and young child feeding practices: Definitions and measurement methods. Geneva: World Health Organization (2021).

[34] Wake AD. Prevalence of minimum meal frequency practice and its associated factors among children aged 6 to 23 months in Ethiopia: a systematic review and meta-analysis. Glob Pediatr Health. (2021) 8:2333794X2110261. doi: 10.1177/2333794X211026184 PMC8226363 34235233

[35] Bitew FH. Spatio-temporal inequalities and predictive models for determinants of undernutrition among women and children in Ethiopia. San Antonio, TX: The University of Texas (2020).

[36] Tessema ZT Tamirat KS. Determinants of high-risk fertility behavior among reproductive-age women in Ethiopia using the recent Ethiopian Demographic Health Survey: a multilevel analysis. Trop Med Health. (2020) 48:93. doi: 10.1186/s41182-020-00280-1 33292871 PMC7697365

[37] Belay AT Fenta SM Birhan Biresaw H Abebaw Moyehodie Y Melkam Yelam M Mekie M. The magnitude of optimal antenatal care utilization and its associated factors among pregnant women in South Gondar zone, Northwest Ethiopia: a cross-sectional study. Int J Reprod Med. (2022) 2022:1415247. doi: 10.1155/2022/1415247 36092776 PMC9463004

[38] Sommet N Morselli D. Keep calm and learn multilevel logistic modeling: a simplified three-step procedure using Stata, R, mplus, and SPSS. Int Rev Soc Hist. (2017) 30:203–18. doi: 10.5334/irsp.90

[39] Goldstein H. Multilevel statistical models. New York, NY: John Wiley & Sons (2011).

[40] Snijders TA Bosker R. Multilevel analysis: An introduction to basic and advanced multilevel modeling. Thousand Oaks: Sage Publishing (2011).

[41] Penny W Holmes A. Random effects analysis. Stat Para Map. (2007) 156:165. doi: 10.1016/B978-012372560-8/50012-7

[42] Borenstein M Hedges LV Higgins JP Rothstein HR. A basic introduction to fixed-effect and random-effects models for meta-analysis. Res Synth Methods. (2010) 1:97–111. doi: 10.1002/jrsm.12 26061376

[43] Rodriguez G Elo I. Intra-class correlation in random-effects models for binary data. Stata J. (2003) 3:32–46. doi: 10.1177/1536867X0300300102

[44] Merlo J Chaix B Ohlsson H Beckman A Johnell K Hjerpe P. A brief conceptual tutorial of multilevel analysis in social epidemiology: using measures of clustering in multilevel logistic regression to investigate contextual phenomena. J Epidemiol Community Health. (2006) 60:290–7. doi: 10.1136/jech.2004.029454 16537344 PMC2566165

[45] Tesema GA Mekonnen TH Teshale AB. Individual and community-level determinants, and spatial distribution of institutional delivery in Ethiopia, 2016: spatial and multilevel analysis. PLoS One. (2020) 15:e0242242. doi: 10.1371/journal.pone.0242242 33180845 PMC7660564

[46] Peeters A Blake MR. Socioeconomic inequalities in diet quality: from identifying the problem to implementing solutions. Curr Nutr Rep. (2016) 5:150–9. doi: 10.1007/s13668-016-0167-5

[47] Aminata Y Sidikiba S Franck G Djiba D Anne M Ousmane O. Minimum acceptable diet intake and associated factors among children aged 6–23 months in Guinea: a multilevel analysis of secondary data. BMC Public Health. (2024) 2:684. doi: 10.5281/hra.v2i2.5261

[48] Gautam KP Adhikari M Khatri RB Devkota MD. Determinants of infant and young child feeding practices in Rupandehi, Nepal. BMC Res Notes. (2016) 9:135. doi: 10.1186/s13104-016-1956-z 26936368 PMC4776375

[49] Pranita RF Briawan D Ekayanti I Triwinarto AJ. Minimum acceptable diet and its associated factors among children aged 6–23 months in Indonesia. Jurnal Gizi dan Pangan. (2023) 18:1–10. doi: 10.25182/jgp.2023.18.1.1-10

[50] Shaun MMA Nizum MWR Munny SJH. Determinants of meeting the minimum acceptable diet among children aged 6 to 23 months in Bangladesh: evidence from a national representative cross-sectional study. Heliyon. (2023) 9:e17560. doi: 10.1016/j.heliyon.2023.e17560 37416681 PMC10320174

[51] Belay DG Taddese AA Gelaye KA. Minimum acceptable diet intake and its associated factors among children age at 6–23 months in sub-Saharan Africa: a multilevel analysis of the sub-Saharan Africa demographic and health survey. BMC Public Health. (2022) 22:684. doi: 10.1186/s12889-022-12966-8 35392871 PMC8991979

[52] Teshome F Tadele A. Trends and determinants of minimum acceptable diet intake among infant and young children aged 6–23 months in Ethiopia: a multilevel analysis of Ethiopian demographic and health survey. BMC Nutr. (2022) 8:44. doi: 10.1186/s40795-022-00533-8 35513888 PMC9069791

[53] Desmennu A Leshi O. Exclusive breastfeeding, minimum acceptable diet, and nutritional status of children: The case of selected West African Countries. Curr Dev Nutr. (2022) 6:560. doi: 10.1093/cdn/nzac060.018

[54] Ba D Ssentongo P Lekoubou A Holland N Maiga M Gao X. Prevalence and determinants of minimum acceptable diet among children aged 6–23 months in sub-Saharan Africa: The Demographic and Health Surveys, 2019–2020. Front Public Health. (2022) 6:884. doi: 10.1093/cdn/nzac067.004 PMC9445207 36081474

[55] Victor R Baines SK Agho KE Dibley M. Factors associated with inappropriate complementary feeding practices among children aged 6–23 months in Tanzania. Matern Child Nutr. (2014) 10:545–61. doi: 10.1111/j.1740-8709.2012.00435.x 22925557 PMC6860229

[56] Gatica-Domínguez G Neves PA Barros AJ Victora CG. Complementary feeding practices in 80 low- and middle-income countries: prevalence of and socioeconomic inequalities in dietary diversity, meal frequency, and dietary adequacy. J Nutr. (2021) 151:1956–64. doi: 10.1093/jn/nxab088 33847352 PMC8245881

[57] Worku MG Alamneh TS Tesema GA Alem AZ Tessema ZT Liyew AM. Minimum acceptable diet feeding practice and associated factors among children aged 6–23 months in East Africa: a multilevel binary logistic regression analysis of 2008–2018 demographic health survey data. Arch Public Health. (2022) 80:127. doi: 10.1186/s13690-022-00882-7 35484576 PMC9047376

[58] Jaleel A Surya Goud C Shankar S Venkatesh K. Nourishing the future: exploring the factors influencing minimum diet diversity and minimum acceptable diet among Indian children aged 6–23 months. J Public Health (Berl.). (2023) 1–13. doi: 10.1007/s10389-023-02085-y

[59] Guirindola MO Maniego MLV Silvestre CJ Acuin CC. Determinants of meeting the minimum acceptable diet among Filipino children aged 6–23 months. Philippine J Sci. (2018) 147:75–89.

[60] Gregory CA Mancino L Coleman-Jensen A. Food security and food purchase quality among low-income households: Findings from the National Household Food Acquisition and purchase survey (food APS). Washington, DC: Economic Research Service (2019).

[61] Ng CS Dibley MJ Agho KE. Complementary feeding indicators and determinants of poor feeding practices in Indonesia: a secondary analysis of 2007 demographic and health survey data. Public Health Nutr. (2012) 15:827–39. doi: 10.1017/S1368980011002485 22014663

[62] Locks LM Pandey PR Osei AK Spiro DS Adhikari DP Haselow NJ. Using formative research to design a context-specific behaviour change strategy to improve infant and young child feeding practices and nutrition in Nepal. Matern Child Nutr. (2015) 11:882–96. doi: 10.1111/mcn.12032, PMID: 23557321 PMC6860308

[63] Khan AM Kayina P Agrawal P Gupta A Kannan AT. A study on infant and young child feeding practices among mothers attending an urban health center in East Delhi. Indian J Public Health. (2012) 56:301–4. doi: 10.4103/0019-557X.106420 23354143

[64] Belew AK Ali BM Abebe Z Dachew BA. Dietary diversity and meal frequency among infant and young children: a community based study. Ital J Pediatr. (2017) 43:1–10. doi: 10.1186/s13052-017-0384-6 28810887 PMC5558775

[65] Aemro M Mesele M Birhanu Z Atenafu A. Dietary diversity and meal frequency practices among infant and young children aged 6–23 months in Ethiopia: a secondary analysis of Ethiopian demographic and health survey 2011. J Nutr Metab. (2013) 2013:1–8. doi: 10.1155/2013/782931, PMID: 24455218 PMC3878383

[66] Tegegne M Sileshi S Benti T Teshome M Woldie H. Factors associated with minimal meal frequency and dietary diversity practices among infants and young children in the predominantly agrarian society of bale zone, Southeast Ethiopia: a community based cross sectional study. Arch Public Health. (2017) 75:1–11. doi: 10.1186/s13690-017-0216-6 29158896 PMC5682638

[67] Joshi N Agho KE Dibley MJ Senarath U Tiwari K. Determinants of inappropriate complementary feeding practices in young children in Nepal: secondary data analysis of demographic and health survey 2006. Matern Child Nutr. (2012) 8:45–59. doi: 10.1111/j.1740-8709.2011.00384.x, PMID: 22168518 PMC6860874

[68] Fahmida U Santika O Kolopaking R Ferguson E. Complementary feeding recommendations based on locally available foods in Indonesia. Food Nutr Bull. (2014) 35:S174–9. doi: 10.1177/15648265140354S302 25639135

[69] Tomedi A Rohan-Minjares F McCalmont K Ashton R Opiyo R Mwanthi M. Feasibility and effectiveness of supplementation with locally available foods in prevention of child malnutrition in Kenya. Public Health Nutr. (2012) 15:749–56. doi: 10.1017/S1368980011002217, PMID: 21896234

[70] Khanal V Sauer K Zhao Y. Determinants of complementary feeding practices among Nepalese children aged 6–23 months: findings from demographic and health survey. BMC Pediatr. (2011) 2013:131. doi: 10.1186/1471-2431-13-131 PMC3766108 23981670

[71] Abdurahman AA Chaka EE Bule MH Niaz KJH. Magnitude and determinants of complementary feeding practices in Ethiopia: a systematic review and meta-analysis. Heliyon. (2019) 5:e01865. doi: 10.1016/j.heliyon.2019.e01865 31317077 PMC6611936

[72] Gewa CA Leslie TF. Distribution and determinants of young child feeding practices in the East African region: demographic health survey data analysis from 2008-2011. J Health Popul Nutr. (2015) 34:1–14. doi: 10.1186/s41043-015-0008-y 26825452 PMC5026023

[73] Abebe H Gashu M Kebede A Abata H Yeshaneh A Workye H. Minimum acceptable diet and associated factors among children aged 6–23 months in Ethiopia. Ital J Pediatr. (2021) 47:1–10. doi: 10.1186/s13052-021-01169-3 34717712 PMC8557568

[74] Kambale RM Ngaboyeka GA Kasengi JB Niyitegeka S Cinkenye BR Baruti A. Minimum acceptable diet among children aged 6–23 months in south Kivu, Democratic Republic of Congo: a community-based cross-sectional study. BMC Pediatr. (2021) 21:239. doi: 10.1186/s12887-021-02713-0 34011304 PMC8132412

[75] Monterrosa EC Frongillo EA Drewnowski A de Pee S Vandevijvere S Bulletin N. Sociocultural influences on food choices and implications for sustainable healthy diets. Food Nutr Bull. (2020) 41:59S–73S. doi: 10.1177/0379572120975874 33356592

[76] Dammann KW Smith C. Factors affecting low-income women's food choices and the perceived impact of dietary intake and socioeconomic status on their health and weight. J Nutr Educ Behav. (2009) 41:242–53. doi: 10.1016/j.jneb.2008.07.003 19508929

[77] Incoom ABM Adjei KA Odai SN. Rainfall variabilities and droughts in the savannah zone of Ghana from 1960-2015. Scientific African. (2020) 10:e00571. doi: 10.1016/j.sciaf.2020.e00571

[78] Adanu SK Mensah FK Adanu SK. Enhancing environmental integrity in the northern savanna zone of Ghana: a remote sensing and GIS approach. J Environ Earth Sci. (2013) 3:67–77.

[79] Taren D Chen J. A positive association between extended breast-feeding and nutritional status in rural Hubei Province, People's Republic of China. Am J Clin Nutr. (1993) 58:862–7. doi: 10.1093/ajcn/58.6.862, PMID: 8249868

[80] Gebretsadik S Gabreyohannes E. Determinants of under-five mortality in high mortality regions of Ethiopia: an analysis of the 2011 Ethiopia demographic and health survey data. Int J Popul Res. (2016) 2016:1–7. doi: 10.1155/2016/1602761

