# Supplementary material for: Minimum acceptable diet use and its associated factors among children aged 6–23 in Ghana: a mixed effect analysis using Ghana Demographic and Health Survey
Source: Front Public Health. 2024 Sep 4;12:1402909. doi: 10.3389/fpubh.2024.1402909 (PMC11408242; doi:10.3389/fpubh.2024.1402909)
Supplement: Supplementary file 1 [file Table_1.docx]

**Appendix**

**Supplementary table 1. Bivariate analysis of the association between covariates and minimum acceptable diet among children aged 6-23 months in Ghana**

**Bivariate analysis**

After the chi-square test, the association between each independent variable and the outcome variable, an independent variable with a p-value <0.05, was taken to bivariate analysis as follows: Independent variables with a p-value < 0.2 were also considered for multivariable analysis.

| **Variable** | **Response** | **MAD use**  **COR (95% CI)** | **p-value** |
| --- | --- | --- | --- |
| Mothers age | 15-19  20-34  35-49 | 0.51(0.33-0.80)  1  1.12 (0.91-1.39) | 0.03  0.289 |
| Mothers education | No education  Primary  Secondary  Higher | 1  1.12 (0.82-1.51)  1.30 (1.03-1.64)  3.20 (2.23-4.58) | 0.475  0.029  0.000 |
| Mother working | Yes  No | 1  0.62 (49-0.78) | 0.000 |
| Fathers education | No education  Primary  Secondary  Higher | 1  1.32 (0.93-1.90)  1.14 (0.89-1.47)  2.57 (1.88-3.52) | 0.124  0.293  0.000 |
| Number of under five | 1  2  ≥ 5 | 1.46 (1.11-1.92)  1.34 (1.01-1.77)  1 | 0.007  0.039 |
| Household head sex | Male  Female | 0.80 (0.65-0.98)  1 | 0.035 |
| Wealth index | Poor  Middle  Rich | 1  1.21(0.93-1.57)  1.97 (1.56-2.48) | 0.151  0.000 |
| Child age | 6-8  9-11  12-23 | 1  2.10 (1.48-3.00)  2.57 (1.92-3.44) | 0.000  0.000 |
| Currently breast feed | Yes  No | 1.31 (1.05-1.64)  1 | 0.016 |
| Plurality | Single  Multiple | 1.98 (1.19-3.31)  1 | 0.009 |
| Media exposure | Yes  No | 1  0.68 (0.54-0.88 | 0.03 |
| ANC visit | Optimal  Non optimal | 1  0.69 (0.5-0.94) | 0.018 |
| PNC visit | Yes  No | 1.36 (1.12-1.65)  1 | 0.002 |
| Counsel on breast feed | Yes  No | 1.32 (1.05-1.64)  1 | 0.016 |
| Place of delivery | Home  Health facility | 1  1.21 (0.91-1.62) | 0.18 |
| Place of residence | Urban  Rural | 1.45 (1.16-1.80)  1 | 0.001 |
| Distance to health facility | Big problem  No problem | 1  0.26 (0.076-0.45) | 0.006 |
| Community poverty level | High  Low | 0.28 (0.12-0.45)  1 | 0.001 |
| Community women illiteracy | High  Low | 0.23 (0.06-0.40)  1 | 0.008 |
| Community media exposure | High  Low | 0.25 (0.08-0.43)  1 | 0.004 |
| Region | Western  Central  Greater Accra  Volta  Eastern  Ashanti  Western north  Ahafo  Bono  Bono east  Oti  Northern  Savannah  North east  Upper east  Upper west | 1  0.30 (0.19 0.79)  0.07 (0.45 0.60)  -0.11 (0.65 0.42)  -0.52 (1.10 0.06)  -0.07 (0.56 0.41)  -0.20 90.74 0.33)  -0.22 90.74 0.31)  -0.23 (0.78 0.31)  -0.53 (1.03 0.03)  0.11 (0.37 0.60)  -0.54 (1.00 0.08)  -1.06 (1.59 0.52)  0.31 (0.13 0.76)  0.04 (0.44 0.52)  -0.07 (0.55 0.42) | 1  0.23  0.78  0.68  0.08  0.77  0.45  0.42  0.40  0.04  0.65  0.02  0.00  0.17  0.88  0.79 |
